# Predictive factors for severe long-term chronic kidney disease after acute kidney injury requiring renal replacement therapy in critically ill patients: an ancillary study of the ELVIS randomized controlled trial

**DOI:** 10.1186/s13054-022-04233-4

**Published:** 2022-11-29

**Authors:** Edouard Soum, Jean-François Timsit, Stephane Ruckly, Didier Gruson, Emmanuel Canet, Kada Klouche, Laurent Argaud, Maïté Garrouste-Orgeas, Christophe Mariat, François Vincent, Sophie Cayot, Michael Darmon, Julien Bohé, Carole Schwebel, Lila Bouadma, Claire Dupuis, Bertrand Souweine, Alexandre Lautrette

**Affiliations:** 1 Medical Intensive Care Unit, Intensive Care Medicine, Montpied Teaching Hospital, 54 Rue Montalembert, BP69, 63003 Clermont-Ferrand, Cedex 1, France; 2 Medical Intensive Care Unit, Albert Michallon Teaching Hospital, Grenoble, France; 3 Medical Intensive Care Unit, Bichat-Claude Bernard Teaching Hospital, Paris, France; 4 ICURESEARCH, Paris, France; 5 Medical Intensive Care Unit, Pellegrin Teaching Hospital, Bordeaux, France; 6 Medical Intensive Care Unit, Saint Louis Teaching Hospital, Paris, France; 7 Medical Intensive Care Unit, Lapeyronie Teaching Hospital, Montpellier, France; 8 Medical Intensive Care Unit, Edouard Herriot Teaching Hospital, Lyon, France; 9 Medical Unit, French British Hospital, Levallois-Perret, France; 10 Nephrology and Critical Care Unit, Nord Teaching Hospital, Saint Etienne, France; 11 Medical Intensive Care Unit, Avicenne Teaching Hospital, Paris, France; 12 Department of Anaesthesiology and Critical Care Medicine, Estaing Teaching Hospital, Clermont-Ferrand, France; 13 Medical Intensive Care Unit, Nord Teaching Hospital, Saint Etienne, France; 14 Medical Intensive Care Unit, Groupement Hospitalier Sud, Hospices Civils de Lyon, Pierre Bénite, Lyon, France; 15 LMGE (Laboratoire Micro-Organismes: Génome et Environnement), UMR CNRS 6023, Université Clermont Auvergne, Clermont-Ferrand, France

**Keywords:** Renal replacement therapy, Acute kidney injury, Critically ill patient, ICU, Outcome, Chronic kidney disease

## Abstract

**Background:**

Acute kidney injury (AKI) requiring renal replacement therapy (RRT) is a serious complication in the ICU that results in increased mortality and risk of chronic kidney disease (CKD). Some studies suggest RRT modality may have an impact on long-term renal recovery after AKI. However, other predictive factors of severe long-term CKD in ICU patients with AKI requiring RRT are unknown.

**Methods:**

We performed an ancillary study of the multicenter ELVIS trial in the population with AKI requiring RRT. Patients alive 3 months after RRT initiation were eligible. Serum creatinine levels available at 3, 6 and 12 months and 3 and 5 years were recorded. CKD stage was determined according to the glomerular filtration rate as estimated by the CKD-EPI formula. At each timepoint, two groups of patients were compared, a no/mild CKD group with normal or mildly to moderately decreased renal function (stages 1, 2 and 3 of the international classification) and a severe CKD group (stages 4 and 5). Our objective was to identify predictive factors of severe long-term CKD.

**Results:**

Of the 287 eligible patients, 183 had follow-up at 3 months, 136 (74.3%) from the no/mild CKD group and 47 (25.7%) from the severe CKD group, and 122 patients at 5 years comprising 96 (78.7%) from the no/mild CKD group and 26 (21.3%) from the severe CKD group. Multivariate analysis showed that a long RRT period was associated with severe CKD up to 12 months (OR_M12_ = 1.03 95% CI [1.02–1.05] per day) and that a high SOFA score at the initiation of RRT was not associated with severe CKD up to 5 years (OR_M60_ = 0.85 95% CI [0.77–0.93] per point).

**Conclusion:**

Severe long-term CKD was found in 21% of ICU survivors who underwent RRT for AKI. The duration of the RRT in AKI patients was identified as a new predictive factor for severe long-term CKD. This finding should be taken into consideration in future studies on the prognosis of ICU patients with AKI requiring RRT.

*Trial registration* ELVIS trial was registered with ClinicalTrials.gov, number: NCT00875069 (June 16, 2014), and this ancillary study was registered with ClinicalTrials.gov, number: NCT03302624 (October 6, 2017).

**Supplementary Information:**

The online version contains supplementary material available at 10.1186/s13054-022-04233-4.

## Background

Acute kidney injury (AKI) is a frequent complication in the intensive care unit (ICU) that affects the vital and renal prognosis of patients in the short and long term [1]. ICU patients risk developing chronic kidney disease (CKD) from stage 1 of the KDIGO classification [2, 3]. Several studies have reported an association between increased severity of AKI and an increased risk of developing CKD in the short and long term [2, 4–7]. The need for RRT is the most severe stage of AKI. Studies report that 70–99% of patients surviving after an AKI requiring an RRT recovered renal autonomy at 6 months and one year [6, 8–10]. However, long-term renal function in these patients remains unknown. In addition, the modality of RRT used to treat AKI could have an impact on renal recovery depending on whether it is continuous or intermittent [11]. Some studies suggest that renal recovery after AKI is greater when the RRT modality is continuous rather than intermittent [12, 13]. Other risk factors for severe long-term CKD in ICU patients with AKI requiring RRT are unknown [14]. The identification of these factors is a major public health objective. Indeed, early management of these factors could improve the vital and renal prognosis, in particular by reducing the risk of recourse to chronic dialysis, which is a significant burden on public health expenditure [15].

The aim of our study was to identify predictive factors for severe long-term CKD in ICU patients with AKI requiring RRT.

## Methods

### Study design and data patients

We conducted an observational, retrospective multicenter ancillary study of the randomized ELVIS trial, which assessed the prevention of RRT catheter infections with ethanol lock [16]. The study was approved by the “Est IV” personal protection committee. (IDRCB No. 2017-A01133-50) and declared to the National Commission for Computing and Freedoms (No. 2008449 v 0). It followed the STROBE (STrengthening the Reporting of Observational studies in Epidemiology) recommendations for good clinical research practice (Additional file 2: Appendix 1).

Eligible patients were aged 18 years or over, enrolled in the ELVIS trial, hospitalized in an ICU, had AKI requiring RRT with the placement of a double-lumen dialysis catheter, discharged from the hospital after 28 days and were alive 3 months after RRT initiation. The study population consisted of patients with at least one serum creatinine value available between 3 months and 5 years after RRT initiation. The exclusion criteria were CKD requiring chronic RRT at inclusion in the ELVIS trial and refusal to participate in the study.

Additional data (serum creatinine level and mortality) to those collected prospectively in the ELVIS trial were retrieved from computerized medical records when possible. Otherwise, patients were contacted by telephone. Five contact tests were performed. If the patient could not be contacted, five attempts to contact the family or the attending physician were made. The patient was considered lost to follow-up if none of the information could be collected after five attempted contact. Patients were informed at the beginning of the interview of the aim and nature of the study and were told they could opt not to take part. If they agreed to participate, their responses were collected for analysis and anonymization. When possible, a picture of the blood test with creatinine level was sent by the patient. The data collected were the serum creatinine level available at 3, 6 and 12 months and at 3 and 5 years, the date of the start of dialysis, and the date of kidney transplantation. If there were several serum creatinine levels available at the same timepoint, the lowest level was recorded. The family or the attending physician was contacted for missing data and, when appropriate, the date of death. The clinical variables were those collected during the ELVIS trial. The RRT period was calculated to run from initiation in the ICU to the last day of treatment, which could be in the ICU or after discharge.

The estimated glomerular filtration rate (eGFR) was measured at each timepoint (3, 6 and 12 months, 3 and 5 years) from the serum creatinine level, age, sex and ethnicity (13 patients were of African race/ethnicity) according to the CPK-EPI formula [17]. For each timepoint, the patients were divided into two groups according to the stages of the CKD classification. The “no/mild CKD” group comprised patients with normal renal function (stage 1 of the international CKD classification [18]) or no/mild CKD (stage 2) or moderate CKD (stage 3A and B). The “severe CKD” group comprised patients with severe CKD (stage 4), preterminal (stage 5) or terminal CKD (chronic dialysis), or renal transplant recipients. The patients could change groups (no/mild vs severe) at any timepoint, depending on the CKD stage. Those whose CKD increased in severity were moved from the no/mild CKD group to the severe CKD group. Patients whose CKD decreased in severity were moved from the severe CKD group to the no/mild CKD group.

### Statistical analysis

The values ​​of the continuous variables are expressed as median [interquartile range, IQR]. The values ​​of the categorical variables are expressed as absolute values and percentages. The total population was separated into the no/mild CKD group and the severe CKD group, as previously defined at 3, 6 and 12 months and 3 and 5 years after RRT initiation. Missing CKD data were imputed by taking the most severe CKD stage before or after the timepoint for which data were missing (*n* = 52 at M3, *n* = 76 at M6, *n* = 76 at M12, *n* = 62 at M36 and *n* = 40 at M60).

The variables of the two groups were compared at each timepoint with Student’s test or Mann–Whitney test for the continuous variables and Chi2 or Fisher’s exact test for the categorical variables. The factors associated with severe CKD were then determined with a logistic regression model. The candidate variables for multivariate analysis were selected based on the results of the univariate analysis (*p* ≤ 0.2) and their clinical relevance: age, only intermittent modality for RRT, insulin-dependent diabetes, chronic kidney disease, chronic respiratory failure with oxygen therapy, SOFA score at RRT initiation, RRT period, septic shock, and immunosuppression. Stepwise regression was then used to select the most informative predictor variables to include in the final multivariate model. The interactions between the explanatory variables were carefully checked before being included in the multivariate analysis. Special attention was also paid to multicollinearity. Linearity to the logit for continuous variables was checked with generalized additive models.

A sensitivity analysis was performed to assess a different classification of CKD categories made up of three groups: normal group (stages 1 and 2), moderate CKD group (stages 3A and 3B) and severe CKD group (stages 4 and 5). The results are expressed in terms of odds ratios (OR) without adjustment and 95% confidence intervals (95%CI). Analyses were performed with SAS 9.4 software (SAS Institute; Cary, NC, USA). A *p* value < 0.05 was considered significant.

## Results

### Patient characteristics

**Fig. 1:**
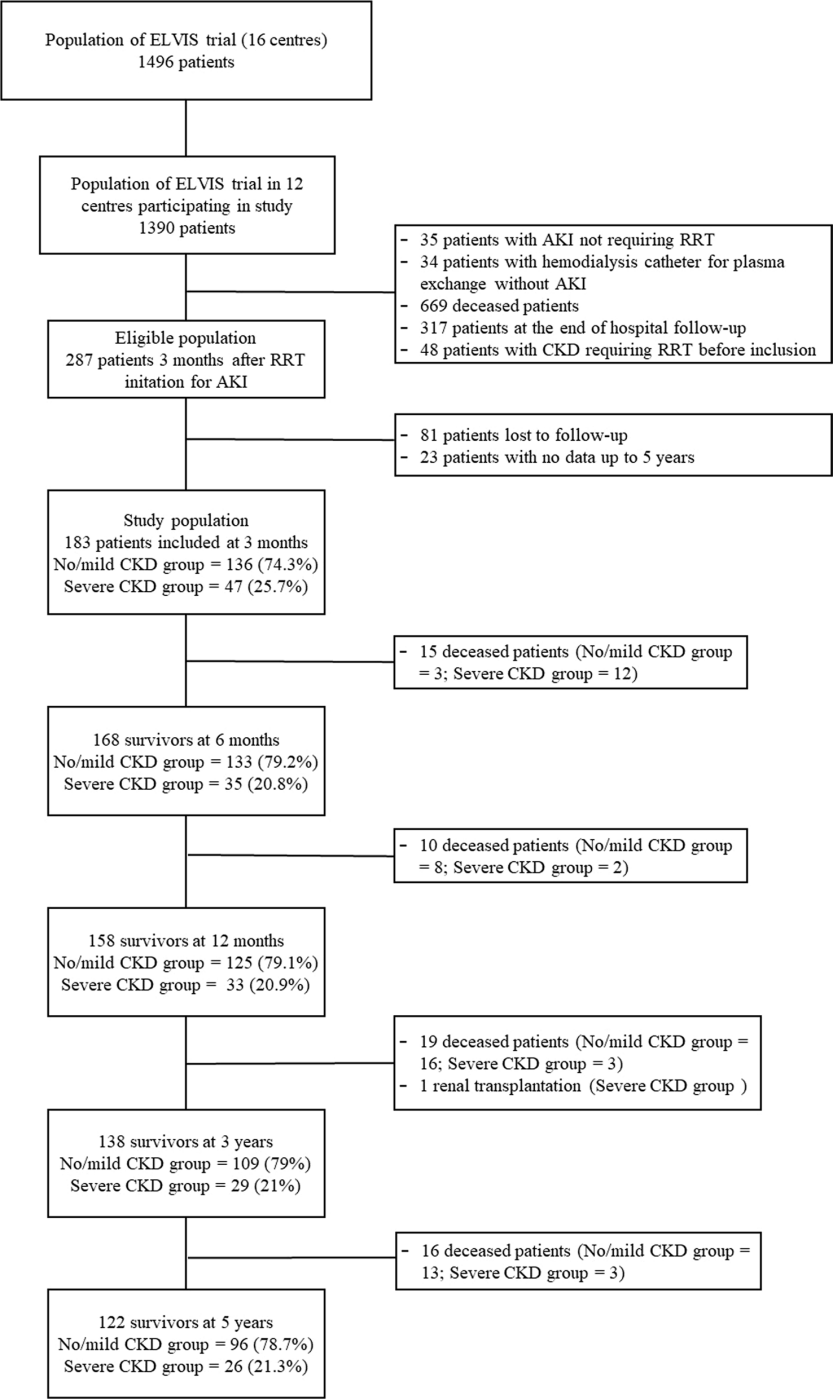
Flowchart. *AKI* acute kidney injury, *CKD* chronic kidney disease

Twelve of the 16 centers involved in the ELVIS trial agreed to participate in our study, providing a pool of 1390 patients out of 1496 that made up the initial inclusion population. Of these 1390 patients, 287 were alive 3 months after RRT initiation for AKI and therefore eligible for the study. At 3 months, 183 patients met the inclusion criteria and constituted the study population (Fig. 1) and 104 patients were excluded for loss to follow-up or because no data were available for up to 5 years, their characteristics are shown in Table 1 and Additional file 1: Table S1, respectively. In the study population, 68 (37%) patients received invasive mechanical ventilation in the ICU, and their median of ICU and hospital length of stays were 21 [13–39] and 61 [44–97] days, respectively.

**Table 1:** Characteristics of study population and comparison between no/mild chronic kidney disease and severe chronic kidney disease groups at 3 months

Variables	Study population	No/mild CKD group	Severe CKD group	OR [IC 95%]	*p* value
Patients, *n*	183	136	47		
Female, *n* (%)	78 (42.6)	58 (42.6)	20 (42.6)	0.99 [0.51–1.95]	0.99
Age, years	62 [54–72]	61 [53.5–69.5]	65 [56–73]	1.02 [0.99–1.04]	0.23
BMI at ICU admission, kg/m^2^	26.9 [23.3–33]	27.5 [23.2–33]	26.1 [23.5–33.2]	0.98 [0.92–1.04]	0.47
SOFA score at RRT initiation, points	14 [11–17]	15 [11–18]	11 [7–15]	0.88 [0.82–0.94]	< 0.01
SAPS II at ICU admission, points	63 [50–80]	64 [51–80]	57 [45–76]	0.98 [0.97–1]	0.09
Intermittent modality for the first RRT, *n* (%)	106 (57.9)	78 (57.4)	28 (59.6)	1.09 [0.56–2.15]	0.79
Only intermittent modality for RRT, *n* (%)	90 (49.2)	65 (47.8)	25 (53.2)	1.14 [0.54–2.47]	0.71
Only continuous modality for RRT, *n* (%)	37 (20.2)	29 (21.3)	8 (17)	0.83 [0.31–2.23]	0.71
RRT period, days	20 [8–42]	18 [7–35]	36 [10–56]	1.02 [1.01–1.03]	< 0.01
RRT sessions, *n*	6 [2–11]	5 [2–10]	7 [3–16]	1.03 [1–1.07]	0.05
Intermittent RRT sessions, *n*	4 [2–8]	4 [2–8]	5 [3–11]	1.04 [0.99–1.08]	0.12
Continuous RRT sessions, *n*	3 [2–7]	3 [2–6]	4.5 [2–12]	1.05 [0.98–1.13]	0.14
Underlying condition					
No comorbidity, *n* (%)	45 (24.6)	30 (22.1)	15 (31.9)	1.66 [0.79–3.46]	0.18
Insulin-dependent diabetes, *n* (%)	24 (13.1)	16 (11.8)	8 (17)	1.54 [0.61–3.87]	0.36
Chronic hypertension, *n* (%)	76 (41.5)	59 (43.4)	17 (36.2)	0.74 [0.37–1.47]	0.39
Hematological malignancy, *n* (%)	18 (9.8)	14 (10.3)	4 (8.5)	0.81 [0.25–2.6]	0.72
Cirrhosis, *n* (%)	13 (7.1)	11 (8.1)	2 (4.3)	0.50 [0.11–2.37]	0.39
Metastatic cancer, *n* (%)	3 (1.6)	2 (1.5)	1 (2.1)	1.46 [0.13–16.44]	0.76
Chronic kidney disease*, *n* (%)	12 (6.6)	5 (3.7)	7 (14.9)	4.58 [1.38–15.24]	0.01
Chronic respiratory failure with oxygen therapy, *n* (%)	6 (3.3)	3 (2.2)	3 (6.4)	3.02 [0.59–15.52]	0.19
Chronic heart failure NYHA III or IV, *n* (%)	21 (11.5)	18 (13.2)	3 (6.4)	0.45 [0.13–1.59]	0.21
Immunosuppression**, *n* (%)	17 (9.3)	10 (7.4)	7 (14.9)	2.20 [0.79–6.17]	0.13
Main symptom at ICU admission					
Septic shock, *n* (%)	57 (31.1)	46 (33.8)	11 (23.4)	0.59 [0.28–1.28]	0.19
Coma, *n* (%)	6 (3.3)	5 (3.7)	1 (2.1)	0.57 [0.06–5]	0.61
Acute respiratory distress, *n* (%)	33 (18)	23 (16.9)	10 (21.3)	1.33 [0.58–3.05]	0.50
Acute kidney injury, *n* (%)	38 (20.8)	26 (19.1)	12 (25.5)	1.45 [0.66–3.17]	0.35

*BMI* Body Mass Index, *SOFA* Sepsis-related Organ Failure Assessment, *SAPS* Simplified Acute Physiology Score, *RRT* Renal replacement therapy, *NYHA* New York Heart Association

*Chronic kidney disease defined as estimated glomerular filtration rate ≤ 60 ml/min/1.73 m^2^

**Immunosuppression defined as patient with immunosuppressor treatment, chemotherapy, radiotherapy, steroids at least 200 mg/d of hydrocortisone or equivalent for at least 3 months, immunodeficiency induced by diseases such as leukemia, lymphoma, AIDS

Follow-up was performed from 3 months on 183 patients comprising 136 (74.3%) from the no/mild CKD group and 47 (25.7%) from the severe CKD group, and at 5 years on 122 patients comprising 96 (78.7%) from the no/mild CKD group and 26 (21.3%) from the severe CKD group (Fig. 1). During the 5 years of follow-up, 60 (32.7%) patients died and one patient had kidney transplantation.

### Study of follow-up of mortality and renal function at 5 years

Comparison of the no/mild CKD group and the severe CKD group by univariate analysis at 3 months, 6 months, 12 months, 3 years and 5 years is shown in Tables 1 (Additional file 1: Tables S2, S3, S4) and 2, respectively. A significant difference between the two groups at least two timepoints was identified in only three variables. The median SOFA score of the no/mild CKD group was 15 at all timepoints and was higher than that of the severe CKD group whose median score was 11. The median durations of the RRT period in the no/mild CKD group at all timepoints were between 18 and 21 days, while those of the severe CKD group were between 27 and 36 days with significant differences between the two groups at 3 and 12 months. CKD comorbidity before inclusion was more frequently found in the severe CKD group at 3 and 12 months.

**Table 2:** Comparison between no/mild chronic kidney disease group and severe chronic kidney disease group at 5 years

Variables	No/mild CKD group	Severe CKD group	OR [IC 95%]	*p* value
Patients, *n*	96	26		
Female, *n* (%)	46 (47.9)	11 (42.3)	0.80 [0.33–1.81]	0.61
Age, years	61 [48.5; 66]	70.5 [62; 76]	1.04 [1.01–1.08]	0.02
BMI at ICU admission, kg/m^2^	28 [24.2; 34]	26.3 [22; 31.8]	0.97 [0.9–1.05]	0.41
SOFA score at RRT initiation, points	15 [11.5; 17.5]	10.5 [7; 14]	0.85 [0.77; 0.93]	< .01
SAPS II at ICU admission, points	61 [51.5; 80]	57 [42; 77]	0.99 [0.96–1.01]	0.24
Intermittent modality for the first RRT, *n* (%)	53 (55.2)	18 (69.2)	1.82 [0.72–4.6]	0.20
Only intermittent modality for RRT, *n* (%)	43 (44.8)	16 (61.5)	1.97 [0.81–4.79]	0.13
Only continuous modality for RRT, *n* (%)	23 (24)	4 (15.4)	0.87 [0.22–3.45]	0.84
RRT period, days	20 [8; 35]	29.5 [10; 45]	1.01 [0.99–1.03]	0.35
RRT sessions, *n*	5 [2; 10]	7.5 [2; 12]	1.02 [0.97–1.07]	0.48
Intermittent RRT sessions, *n*	4 [2; 8]	5 [2; 11]	1.02 [0.96–1.08]	0.52
Continuous RRT sessions, *n*	3 [2; 6]	5 [1; 8]	1.04 [0.93–1.16]	0.46
Underlying condition				
No comorbidity, *n* (%)	23 (24)	10 (38.5)	1.98 [0.79–4.97]	0.14
Insulin-dependent diabetes, *n* (%)	11 (11.5)	5 (19.2)	1.84 [0.58–5.87]	0.30
Chronic hypertension, *n* (%)	37 (38.5)	10 (38.5)	0.99 [0.41–2.43]	0.99
Hematological malignancy, *n* (%)	10 (10.4)	0 (0)		
Cirrhosis, *n* (%)	7 (7.3)	0 (0)		
Metastatic cancer, *n* (%)	1 (1)	0 (0)		
Chronic kidney disease*, *n* (%)	2 (2.1)	2 (7.7)	4.08 [0.78–21.37]	0.10
Chronic respiratory failure with oxygen therapy, *n* (%)	2 (1.8)	3 (10.3)	3.92 [0.52–29.25]	0.18
Chronic heart failure NYHA III or IV, *n* (%)	12 (12.5)	1 (3.8)	0.28 [0.03–2.26]	0.23
Immunosuppression**, *n* (%)	7 (7.3)	4 (15.4)	2.31 [0.62–8.6]	0.21
Main symptom at ICU admission				
Septic shock, *n* (%)	30 (31.3)	7 (26.9)	0.81 [0.31–2.13]	0.67
Coma, *n* (%)	2 (2.1)	1 (3.8)	1.88 [0.16–21.58]	0.61
Acute respiratory distress, *n* (%)	17 (17.7)	6 (23.1)	1.39 [0.49–3.99]	0.54
Acute kidney injury, *n* (%)	18 (18.8)	5 (19.2)	1.03 [0.34–3.11]	0.96

*BMI* Body Mass Index, *SOFA* Sepsis-related Organ Failure Assessment, *SAPS* Simplified Acute Physiology Score, *RRT* Renal replacement therapy, *NYHA* New York Heart Association

*Chronic kidney disease defined as estimated glomerular filtration rate ≤ 60 ml/min/1.73 m^2^

**Immunosuppression defined as patient with immunosuppressor treatment, chemotherapy, radiotherapy, steroids at least 200 mg/d of hydrocortisone or equivalent for at least 3 months, immunodeficiency induced by diseases such as leukemia, lymphoma, and AIDS

Of the 183 patients included in our study, 168 (91.8%) were alive at 6 months, 158 (86.3%) at one year, 138 (75.4%) at 3 years, and 122 (66.6%) at 5 years (Fig. 1).

**Fig. 2:**
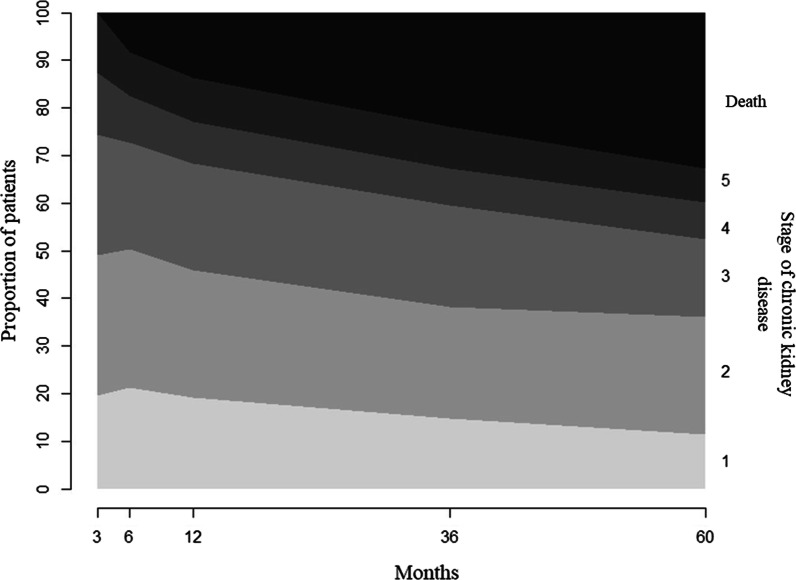
Proportion of patients who died and at different stages of chronic kidney disease over the study period. The no/mild chronic kidney disease group was made up of patients in stages 1, 2 and 3. The severe chronic kidney disease group was made up of patients in stages 4 and 5

The change over time in the proportion of patients in the different stages of CKD and who died is shown in Fig. 2. The proportions of stages 2, 4 and 5 remained stable, while those of stages 1 and 3 decreased.

The results of multivariate analysis at the different timepoints are shown in Table 3. The number of days of the RRT period was independently associated with a risk of developing severe CKD for up to 12 months, while the number of SOFA score points was associated with protection from developing severe CKD for up to 5 years. The sensitivity analysis yielded similar results, showing an association between the RRT period and severe long-term CKD (Additional file 1: Table S5).

**Table 3:** Factors associated with severe chronic kidney disease group (multivariate analysis)

Variables	OR [IC 95%]	*p* value
3 months		
RRT period (per day)	1.03 [1.017–1.048]	< 0.0001
Chronic kidney disease*	4.09 [1.031–16.248]	0.0451
SOFA score at RRT initiation (per point)	0.83 [0.760–0.902]	< 0.0001
6 months		
RRT period (per days)	1.02 [1.004–1.036]	0.0156
SOFA score at RRT initiation (per point)	0.83 [0.765–0.911]	< 0.0001
12 months		
Age	1.04 [1.006–1.081]	0.0215
RRT period (per day)	1.03 [1.011–1.051]	0.0017
SOFA score at RRT initiation (per point)	0.82 [0.742–0.900]	< 0.0001
3 years		
SOFA score at RRT initiation (per point)	0.85 [0.777–0.928]	0.0003
5 years		
SOFA score at RRT initiation (per point)	0.85 [0.771–0.935]	0.0009

*RRT* renal replacement therapy, *SOFA* Sepsis-related Organ Failure Assessment

*Chronic kidney disease defined as estimated glomerular filtration rate ≤ 60 ml/min/1.73 m^2^

## Discussion

Our study shows that a long RRT period for AKI in the ICU is a risk factor for developing severe long-term CKD. In contrast, a high SOFA score at RRT initiation was associated with a lower risk of developing severe CKD in the long term. These associations are reported here for the first time.

It is now well established that AKI is a risk factor for the development of, or progression to, CKD [1, 3, 5–7, 14, 19, 20]. Among the features of RRT used in ICU patients with AKI, only the modality of RRT has been suggested as a risk factor for CKD. In a meta-analysis, Schneider et al. showed that among AKI survivors, initial treatment with intermittent RRT can be associated with higher rates of dialysis dependence than with continuous RRT [13]. Sun et al. showed that continuous RRT was associated with the better renal recovery and a shorter RRT period, but without influencing 60-day mortality [21]. These findings are counterbalanced by those of Vinsonneau et al. which showed that there was no difference in mortality (31.5–32.6% *p* = 0.98) or recovery of renal function (10% vs 7% *p* = 0.5) between continuous and intermittent RRT [22]. Finally, Truche et al. showed that the modality of RRT did not significantly impact mortality and renal recovery at 6 months [23]. However, this last study suggested mortality was reduced with the continuous modality in the subpopulation of ICU patients with positive fluid balance, and with the intermittent modality in the subpopulation of patients without hemodynamic instability. In our study, there was an association between a long period of RRT during AKI and severe CKD up to 1 year. Because of the small size of the severe CKD group at 3 and 5 years, it was not possible to achieve the statistical power needed to show a significant difference after 1 year. Few studies have assessed the impact of AKI duration in the ICU on long-term renal function. Mehta et al. showed in a meta-analysis that a long duration of AKI was a risk of developing CKD [24]. To our knowledge, our study is the first to show that a long period of RRT is associated with a higher risk of severe CKD in the long term. There are several possible explanations for this observation. It may be that the period of AKI during which RRT is required, is associated with the severity of renal lesions. Several studies have shown that the severity of an AKI is a risk factor for developing CKD [4, 5]. In addition, it has been shown that RRT exposes patients to per-procedure arterial hypotension, which is sometimes undetected and could cause ischemic kidney damage [25]. Such ischemic lesions can contribute to the delay of renal recovery, which would promote the fibrotic cellular processes leading to CKD [26]. It seems that RRT is both the consequence of the severity and a factor in the prolongation of the renal lesional process and thereby prevents optimal renal recovery. Many other factors such as hypertension, diabetes, and older age are involved in the development and progression of CKD [3]. Some of our included patients had risk factors that have contributed to the development of severe CKD.

Our study showed an intuitively surprising association between a high SOFA score [27] and the development of long-term low-severity CKD. This association persisted throughout the duration of the follow-up, i.e., 5 years after the AKI. There is no pathophysiological relationship to explain this finding. It could be due to a selection bias in the study population. Patients who had a high organ failure score either died (most often within 3 months) and therefore were not included in the study, or survived without renal sequelae. The two findings of our study suggest that ICU patients who had an RRT period of less than 20 days and who survived despite a significant number of organ failures, had a good renal recovery in the long term. The AKI could mainly result from other organ failures. In survivors, the recovery of these failures leads to a renal recovery. Otherwise, the comorbidities “chronic hypertension” and “insulin-dependent diabetes” were not associated with the severe CKD group, while these comorbidities are well-established risk factors for CKD. Only 13% of patients suffered from insulin-dependent diabetes and 41% suffered from chronic hypertension, so our study might be underpowered to show a difference in the impact of these comorbidities between the two CKD groups. Our results should be taken into consideration in future studies on the prognosis of ICU patients treated with RRT. It is possible that in addition to the modality of RRT, its duration could have an impact on long-term renal recovery and therefore on patient prognosis.

Our study has several limitations. First, the duration of the RRT period depends on when it was initiated and on the weaning criteria. There is currently no international consensus regarding the criteria for the initiation and weaning of RRT, and hence our findings were not affected by a modification of these criteria. The ELVIS trial [16] was carried out before several randomized controlled trials that suggested delaying the initiation of RRT since 40 to 50% of patients who have severe AKI achieve rapid renal recovery, which avoids RRT with no impact on mortality [28, 29]. However, we do not believe that the new suggested criteria for the initiation of RRT would change our results because the duration of RRT in these trials was very short, from 2 to 4 days, which suggests that AKI was less severe than in our study probably owing to a different case mix. However, almost all of the patients included in our study had a 4-point renal SOFA criterion, which corresponds to less than 200 ml/24 h of diuresis or serum creatinine > 440 µmol/L, whereas mean serum creatinine level in the trial participants at the initiation of RRT was less than 300 µmol/L [28–30]. It is therefore unlikely that our study included patients who could have avoided RRT. Similarly, the impact of modifying RRT methods is unlikely, given the absence of any major publications leading to new recommendations for changing practices. Second, the design of our study did not make it possible to ensure that all creatinine levels were measured at each timepoint. Twenty-two patients had a serum creatinine value available between 3 months and 5 years and were alive at 5 years. However, our choice of missing data imputation did not adversely affect the risk of severe CKD because the imputed CKD stage was equal to the more severe of the two stages before or after the timepoint for which data were missing. Thus, our imputation methodology could have identified severe CKD before it actually occurred. Some elderly patients improved their renal function between 3 and 6 months or even up to 1 year and hence changed CKD stage accordingly. We assume that the renal function of the elderly patients took several months to be fully recovered because of the structural and functional changes associated with the aging kidney [31]. Moreover, the baseline of serum creatinine level before ICU admission was not collected in the ELVIS database. Only the variable eGFR ≤ 60 ml/min/1.73 m^2^ was collected, which may have been estimated with means other than CPK-EPI, and this could explain why some of these patients were in the no/mild group. We assessed the occurrence of CKD after AKI requiring RRT and not the renal recovery of patients. Third, generalizing our findings to all critically ill patients requiring RRT for AKI should be cautious because of the lack of information on a significant number of patients who were lost of follow-up or did not have bloodwork at the different timepoints assessed in the study.

## Conclusion

Severe long-term CKD is found in one-fifth of ICU survivors treated with RRT for AKI. The duration of the RRT period has been identified as a risk factor for developing severe CKD in the long term. Our findings should be considered in future studies on the prognosis of ICU patients treated with RRT.

## Supplementary Information

**Additional file 1: Table S1.** Characteristics of patients lost to follow-up or with no data up to 5 years. **Table S2.** Comparison between no/mild chronic kidney disease group and severe chronic kidney disease group at 6 months. **Table S3.** Comparison between no/mild chronic kidney disease group and severe chronic kidney disease group at 12 months. **Table S4.** Comparison between no/mild chronic kidney disease group and severe chronic kidney disease group at 3 years. **Table S5.** Factors associated with severe chronic kidney disease group (multivariate logistic ordinal analysis) when the classification is based on three groups (stages 1–2; stages 3A–3B, stages 4–5).

**Additional file 2: Appendix 1.** STROBE checklist.

## Data Availability

The data that support the findings of this study are available from the corresponding author upon reasonable request.

## Abbreviations

AKI: Acute kidney injury
CKD: Chronic kidney disease
eGFR: Estimated glomerular filtration rate
RRT: Renal replacement therapy
SOFA: Sepsis-related Organ Failure Assessment
