# Supplementary material for: Predictive factors for severe long-term chronic kidney disease after acute kidney injury requiring renal replacement therapy in critically ill patients: an ancillary study of the ELVIS randomized controlled trial
Source: Crit Care. 2022 Nov 29;26:367. doi: 10.1186/s13054-022-04233-4 (PMC9706988; doi:10.1186/s13054-022-04233-4)
Supplement: Supplementary file 1 — Additional file 1: Table S1. Characteristics of patients lost to follow-up or with no data up to 5 years. Table S2. Comparison between no/mild chronic kidney disease group and severe chronic kidney disease group at 6 months. Table S3. Comparison between no/mild chronic kidney disease group and severe chronic kidney disease group at 12 months. Table S4. Comparison between no/mild chronic kidney disease group and severe chronic kidney disease group at 3 years. Table S5. Factors associated with severe chronic kidney disease group (multivariate logistic ordinal analysis) when the classification is based on three groups (stages 1–2; stages 3A–3B, stages 4–5). [file 13054_2022_4233_MOESM1_ESM.docx]

**Table S1:** Characteristics of patients lost to follow-up or with no data up to 5 years

| Variables |  |
| --- | --- |
| Patients, n | 104 |
| Female, n (%) | 39 (37.5) |
| Age, years | 64 [55 – 74] |
| BMI at ICU admission, kg/m^2^ | 27.1 [23.6 – 32.9] |
| SOFA score at RRT initiation , points | 14 [9 – 17] |
| SAPS II at ICU admission, points | 59.5 [48.5 – 74.5] |
| Intermittent modality for the first RRT, n (%) | 60 (57.7) |
| Only intermittent modality for RRT, n (%) | 47 (45.2) |
| Only continuous modality for RRT, n (%) | 21 (20.2) |
| RRT period, days | 34 [13.5 – 49] |
| RRT sessions, n | 5 [2 – 10] |
| Intermittent RRT sessions, n | 4 [2 – 10] |
| Continuous RRT sessions, n | 3 [1 – 5] |
| Underlying condition |  |
| No comorbidity, n (%) | 23 (22.1) |
| Insulin-dependent diabetes, n (%) | 11 (10.6) |
| Chronic hypertension, n (%) | 67 (64.4) |
| Hematological malignacy, n (%) | 8 (7.7) |
| Cirrhosis, n (%) | 1 (1) |
| Metastatic cancer, n (%) | 3 (2.9) |
| Chronic kidney disease*, n (%) | 9 (8.7) |
| Chronic respiratory failure with oxygen therapy, n (%) | 3 (2.9) |
| Chronic heart failure NYHA III or IV, n (%) | 14 (13.5) |
| Immunosuppression**, n (%) | 4 (3.8) |
| Main symptom at ICU admission |  |
| Septic shock, n (%) | 35 (33.7) |
| Coma, n (%) | 6 (5.8) |
| Acute respiratory distress, n (%) | 22 (21.2) |
| Acute kidney injury, n (%) | 26 (25) |
| Invasive mechanical ventilation, n(%) | 36 (34.6) |
| ICU length of stay, days | 20 [10.5 – 35] |
| Hospital length of stay, days | 54 [40.5 – 79.5] |

BMI: Body Mass Index; SOFA : Sepsis-related Organ Failure Assessment ; SAPS : Simplified Acute Physiology Score ; RRT: Renal replacement therapy ; NYHA : New York Heart Association

* : Chronic kidney disease defined as estimated glomerular filtration rate ≤ 60ml/min/1.73m²

** : Immunosuppression defined as patient with immunosuppressor treatment, chemotherapy, radiotherapy, steroids at least 200mg/d of hydrocortisone or equivalent for at least 3 months, immunodeficiency induced by diseases such as leukemia, lymphoma, AIDS

**Table S2:** Comparison between no/mild chronic kidney disease group and severe chronic kidney disease group at 6 months

| Variables | No/mild CKD group | Severe CKD group | OR [IC 95%] | p-value |
| --- | --- | --- | --- | --- |
| Patients, n | 133 | 35 |  |  |
| Female, n (%) | 59 (44.4) | 14 (40) | 0.84 [0.39 - 1.78] | 0.64 |
| Age, years | 61 [52 - 69] | 68 [58 - 76] | 1.03 [1 - 1.06] | 0.04 |
| BMI at ICU admission, kg/m^2^ | 27.4 [23 - 33.1] | 26.3 [22.8 - 31.8] | 0.97 [0.91 - 1.04] | 0.41 |
| SOFA score at RRT initiation , points | 15 [12 - 18] | 11 [7 - 14] | 0.86 [0.79 - 0.93] | <0.01 |
| SAPS II at ICU admission, points | 64 [52 - 80] | 57 [45 - 78] | 0.99 [0.97 - 1.01] | 0.27 |
| Intermittent modality for the first RRT, n (%) | 77 (57.9) | 22 (62.9) | 1.23 [0.57 - 2.65] | 0.60 |
| Only intermittent modality for RRT, n (%) | 65 (48.9) | 20 (57.1) | 1.58 [0.64 - 3.91] | 0.33 |
| Only continuous modality for RRT, n (%) | 27 (20.3) | 7 (20) | 1.33 [0.43 - 4.09] | 0.62 |
| RRT period, days | 20 [7 - 36] | 35 [10 - 47] | 1.01 [1 - 1.03] | 0.11 |
| RRT sessions, n | 5 [2 - 10] | 8 [2 - 16] | 1.03 [0.99 - 1.07] | 0.18 |
| Intermittent RRT sessions, n | 4 [2 - 8] | 5 [2 - 9.5] | 1.02 [0.97 - 1.07] | 0.44 |
| Continuous RRT sessions, n | 3 [2 - 6] | 7 [4 - 13] | 1.09 [1.01 - 1.19] | 0.04 |
| Underlying condition |  |  |  |  |
| No comorbidity, n (%) | 29 (21.8) | 12 (34.3) | 1.87 [0.83 - 4.21] | 0.13 |
| Insulin-dependent diabetes, n (%) | 16 (12) | 7 (20) | 1.83 [0.69 - 4.87] | 0.23 |
| Chronic hypertension, n (%) | 57 (42.9) | 13 (37.1) | 0.79 [0.37 - 1.7] | 0.54 |
| Hemotological malignacy, n (%) | 14 (10.5) | 2 (5.7) | 0.51 [0.11 - 2.38] | 0.40 |
| Cirrhosis, n (%) | 12 (9) | 1 (2.9) | 0.29 [0.04 - 2.36] | 0.25 |
| Metastatic cancer, n (%) | 3 (2.3) | 0 (0) |  |  |
| Chronic kidney disease*, n (%) | 7 (5.3) | 4 (11.4) | 2.32 [0.64 - 8.44] | 0.20 |
| Chronic respiratory failure with oxygen therapy, n (%) | 3 (2.3) | 3 (8.6) | 4.06 [0.78 - 21.08] | 0.20 |
| Chronic heart failure NYHA III or IV, n (%) | 17 (12.8) | 2 (5.7) | 0.41 [0.09 - 1.88] | 0.10 |
| Immunosuppression**, n (%) | 11 (8.3) | 4 (11.4) | 1.43 [0.43 - 4.8] | 0.25 |
| Main symptom at ICU admission |  |  |  |  |
| Septic shock, n (%) | 44 (33.1) | 9 (25.7) | 0.70 [0.3 - 1.62] | 0.41 |
| Coma, n (%) | 5 (3.8) | 1 (2.9) | 0.75 [0.09 - 6.66] | 0.80 |
| Acute respiratory distress, n (%) | 22 (16.5) | 7 (20) | 1.26 [0.49 - 3.25] | 0.63 |
| Acute kidney injury, n (%) | 26 (19.5) | 8 (22.9) | 1.22 [0.5 - 2.99] | 0.67 |

BMI: Body Mass Index; SOFA : Sepsis-related Organ Failure Assessment ; SAPS : Simplified Acute Physiology Score ; RRT: Renal replacement therapy ; NYHA : New York Heart Association

* : Chronic kidney disease defined as estimated glomerular filtration rate ≤ 60ml/min/1.73m²

** : Immunosuppression defined as patient with immunosuppressor treatment, chemotherapy, radiotherapy, steroids at least 200mg/d of hydrocortisone or equivalent for at least 3 months, immunodeficiency induced by diseases such as leukemia, lymphoma, AIDS

**Table S3:** Comparison between no/mild chronic kidney disease group and severe chronic kidney disease group at 12 months

| Variables | No/mild CKD group | Severe CKD group | OR [IC 95%] | p-value |
| --- | --- | --- | --- | --- |
| Patients, n | 125 | 33 |  |  |
| Female, n (%) | 56 (44.8) | 12 (36.4) | 0.70 [0.32 - 1.55] | 0.39 |
| Age, years | 61 [51 - 68] | 66 [61 - 74] | 1.03 [1 - 1.07] | 0.04 |
| BMI at ICU admission, kg/m^2^ | 27.5 [23 - 33.4] | 26.1 [22 - 31.8] | 0.97 [0.91 - 1.04] | 0.47 |
| SOFA score at RRT initiation , points | 15 [12 - 18] | 11 [7 - 14] | 0.85 [0.78 - 0.92] | <0.01 |
| SAPS II at ICU admission, points | 64 [52 - 80] | 57 [44 - 80] | 0.99 [0.97 - 1.01] | 0.30 |
| Intermittent modality for the first RRT, n (%) | 72 (57.6) | 22 (66.7) | 1.47 [0.66 - 3.3] | 0.35 |
| Only intermittent modality for RRT, n (%) | 62 (49.6) | 20 (60.6) | 1.41 [0.56 - 3.54] | 0.46 |
| Only continuous modality for RRT, n (%) | 28 (22.4) | 5 (15.2) | 0.78 [0.23 - 2.65] | 0.69 |
| RRT period, days | 19 [7 - 34] | 35 [15 - 47] | 1.02 [1 - 1.03] | 0.04 |
| RRT sessions, n | 5 [2 - 10] | 7 [3 - 16] | 1.03 [1 - 1.07] | 0.05 |
| Intermittent RRT sessions, n | 4 [2 - 8] | 5 [3 - 11] | 1.04 [0.99 - 1.08] | 0.12 |
| Continuous RRT sessions, n | 3 [2 - 6] | 4.5 [2 - 12] | 1.05 [0.98 - 1.13] | 0.14 |
| Underlying condition |  |  |  |  |
| No comorbidity, n (%) | 26 (20.8) | 11 (33.3) | 1.90 [0.82 - 4.42] | 0.13 |
| Insulin-dependent diabetes, n (%) | 13 (10.4) | 8 (24.2) | 2.76 [1.03 - 7.36] | 0.04 |
| Chronic hypertension, n (%) | 56 (44.8) | 12 (36.4) | 0.70 [0.32 - 1.55] | 0.39 |
| Hematological malignacy, n (%) | 13 (10.4) | 1 (3) | 0.27 [0.03 - 2.14] | 0.21 |
| Cirrhosis, n (%) | 10 (8) | 2 (6.1) | 0.74 [0.15 - 3.56] | 0.71 |
| Metastatic cancer, n (%) | 2 (1.6) | 0 (0) |  |  |
| Chronic kidney disease*, n (%) | 5 (4) | 5 (15.2) | 4.29 [1.16 - 15.82] | 0.03 |
| Chronic respiratory failure with oxygen therapy, n (%) | 3 (2.4) | 3 (9.1) | 4.07 [0.78 - 21.16] | 0.10 |
| Chronic heart failure NYHA III or IV, n (%) | 17 (13.6) | 1 (3) | 0.19 [0.03 - 1.55] | 0.12 |
| Immunosuppression**, n (%) | 10 (8) | 4 (12.1) | 1.59 [0.46 - 5.42] | 0.46 |
| Main symptom at ICU admission |  |  |  |  |
| Septic shock, n (%) | 42 (33.6) | 8 (24.2) | 0.63 [0.26 - 1.52] | 0.31 |
| Coma, n (%) | 3 (2.4) | 2 (6.1) | 2.62 [0.42 - 16.39] | 0.30 |
| Acute respiratory distress, n (%) | 19 (15.2) | 7 (21.2) | 1.50 [0.57 - 3.95] | 0.41 |
| Acute kidney injury, n (%) | 26 (20.8) | 7 (21.2) | 1.02 [0.4 - 2.62] | 0.96 |

BMI: Body Mass Index; SOFA : Sepsis-related Organ Failure Assessment ; SAPS : Simplified Acute Physiology Score ; RRT: Renal replacement therapy ; NYHA : New York Heart Association

* : Chronic kidney disease defined as estimated glomerular filtration rate ≤ 60ml/min/1.73m²

** : Immunosuppression defined as patient with immunosuppressor treatment, chemotherapy, radiotherapy, steroids at least 200mg/d of hydrocortisone or equivalent for at least 3 months, immunodeficiency induced by diseases such as leukemia, lymphoma, AIDS

**Table S4:** Comparison between no/mild chronic kidney disease group and severe chronic kidney disease group at 3 years

| Variables | No/mild CKD group | Severe CKD group | OR [IC 95%] | p-value |
| --- | --- | --- | --- | --- |
| Patients, n | 109 | 29 |  |  |
| Female, n (%) | 50 (45.9) | 12 (41.4) | 0.83 [0.36 - 1.91] | 0.67 |
| Age, years | 61 [51 - 67] | 70 [64 - 75] | 1.04 [1.01 - 1.08] | 0.01 |
| BMI at ICU admission, kg/m^2^ | 27.8 [23.9 - 33.8] | 26.4 [23.5 - 35.1] | 0.99 [0.92 - 1.06] | 0.78 |
| SOFA score at RRT initiation , points | 15 [12 - 18] | 11 [7 - 14] | 0.85 [0.78 - 0.93] | <.01 |
| SAPS II at ICU admission, points | 63 [52 - 80] | 57 [40 - 71] | 0.98 [0.96 - 1] | 0.09 |
| Intermittent modality for the first RRT, n (%) | 60 (55) | 23 (79.3) | 3.13 [1.18 - 8.3] | 0.02 |
| Only intermittent modality for RRT, n (%) | 51 (46.8) | 21 (72.4) | 3.50 [1.1 - 11.1] | 0.03 |
| Only continuous modality for RRT, n (%) | 24 (22) | 4 (13.8) | 1.42 [0.32 - 6.23] | 0.64 |
| RRT period, days | 21 [9 - 37] | 27 [6 - 43] | 1.01 [0.99 - 1.02] | 0.42 |
| RRT sessions, n | 6 [2 - 10] | 4 [1 - 11] | 1.00 [0.95 - 1.05] | 0.95 |
| Intermittent RRT sessions, n | 5 [2 - 8] | 3 [2 - 10] | 1.01 [0.96 - 1.07] | 0.70 |
| Continuous RRT sessions, n | 3 [2 - 6] | 5 [1 - 7.5] | 1.03 [0.91 - 1.16] | 0.68 |
| Underlying condition |  |  |  |  |
| No comorbidity, n (%) | 27 (24.8) | 9 (31) | 1.37 [0.56 - 3.36] | 0.50 |
| Insulin-dependent diabetes, n (%) | 12 (11) | 6 (20.7) | 2.11 [0.72 - 6.21] | 0.18 |
| Chronic hypertension, n (%) | 43 (39.4) | 14 (48.3) | 1.43 [0.63 - 3.26] | 0.39 |
| Hematological malignacy, n (%) | 11 (10.1) | 0 (0) |  |  |
| Cirrhosis, n (%) | 8 (7.3) | 0 (0) |  |  |
| Metastatic cancer, n (%) | 1 (0.9) | 0 (0) |  |  |
| Chronic kidney disease*, n (%) | 3 (2.8) | 3 (10.3) | 4.08 [0.78 - 21.37] | 0.10 |
| Chronic respiratory failure with oxygen therapy, n (%) | 2 (1.8) | 3 (10.3) | 6.17 [0.98 - 38.84] | 0.05 |
| Chronic heart failure NYHA III or IV, n (%) | 15 (13.8) | 2 (6.9) | 0.46 [0.1 - 2.16] | 0.33 |
| Immunosuppression**, n (%) | 8 (7.3) | 4 (13.8) | 2.02 [0.56 - 7.25] | 0.28 |
| Main symptom at ICU admission |  |  |  |  |
| Septic shock, n (%) | 34 (31.2) | 7 (24.1) | 0.70 [0.27 - 1.8] | 0.46 |
| Coma, n (%) | 2 (1.8) | 1 (3.4) | 1.91 [0.17 - 21.84] | 0.60 |
| Acute respiratory distress, n (%) | 18 (16.5) | 7 (24.1) | 1.61 [0.6 - 4.33] | 0.35 |
| Acute kidney injury, n (%) | 21 (19.3) | 7 (24.1) | 1.33 [0.5 - 3.53] | 0.56 |

BMI: Body Mass Index; SOFA : Sepsis-related Organ Failure Assessment ; SAPS : Simplified Acute Physiology Score ; RRT: Renal replacement therapy ; NYHA : New York Heart Association

* : Chronic kidney disease defined as estimated glomerular filtration rate ≤ 60ml/min/1.73m²

** : Immunosuppression defined as patient with immunosuppressor treatment, chemotherapy, radiotherapy, steroids at least 200mg/d of hydrocortisone or equivalent for at least 3 months, immunodeficiency induced by diseases such as leukemia, lymphoma, AIDS

**Table S5:** Factors associated with severe chronic kidney disease group (multivariate logistic ordinal analysis) when the classification is based on three groups (stages 1-2; stages 3A-3B, stages 4-5)

| Variables | OR [IC 95%] | p-value |
| --- | --- | --- |
| 3 months |  |  |
| Age | 1.03 [1.01 – 1.06] | 0.0041 |
| RRT period, (per day) | 1.03 [1.01 – 1.04] | < 0.0001 |
| Chronic kidney disease * | 6.02 [1.66 – 21.83] | 0.0063 |
| SOFA score at RRT initiation, (per point) | 0.88 [0.83 – 0.94] | < 0.0001 |
| 6 months |  |  |
| Age | 1.04 [1.02 – 1.07] | 0.0013 |
| RRT period, (per day) | 1.02 [1.01 – 1.04] | 0.0018 |
| Chronic kidney disease * | 3.45 [1.04 – 11.41] | 0.0429 |
| SOFA score at RRT initiation, (per point) | 0.88 [0.82 – 0.94] | 0.0001 |
| 12 months |  |  |
| Age | 1.05 [1.02 – 1.08] | 0.0005 |
| RRT period, (per day) | 1.02 [1.01 – 1.04] | 0.0014 |
| Chronic kidney disease * | 4.74 [1.30 – 17.27] | 0.0183 |
| SOFA score at RRT initiation, (per point) | 0.88 [0.82 – 0.94] | 0.0002 |
| 3 years |  |  |
| Age | 1.06 [1.03 – 1.08] | 0.0001 |
| RRT period, (per day) | 1.02 [1.01 – 1.04] | 0.0090 |
| SOFA score at RRT initiation, (per point) | 0.87 [0.81 – 0.94] | 0.0001 |
| 5 years |  |  |
| Age | 1.05 [1.02 – 1.08] | 0.0013 |
| RRT period, (per day) | 1.02 [1.00 – 1.03] | 0.0406 |
| SOFA score at RRT initiation, (per point) | 0.89 [0.82 - 0.96] | 0.0024 |

RRT: renal replacement therapy; SOFA : Sepsis-related Organ Failure Assessment ;

*: Chronic kidney disease defined as estimated glomerular filtration rate ≤ 60ml/min/1.73m²
